# Qualitative Exploration of Ultrastructural Effects of Perfluorooctanoic Acid on Carp Gills: Mitochondria-Rich Cells as Candidate Biomarkers of Cytotoxicity

**DOI:** 10.3390/toxics13121020

**Published:** 2025-11-26

**Authors:** Maurizio Manera, Cosma Manera, Giuseppe Castaldelli, Luisa Giari

**Affiliations:** 1 Department of Biosciences, Food and Environmental Technologies, University of Teramo, St. R. Balzarini 1, 64100 Teramo, Italy; 2 General Practitioner, St. Antonio Di Vincenzo 12/2, 40129 Bologna, Italy; cosma.manera@gmail.com; 3 Department of Environmental and Prevention Sciences, University of Ferrara, St. Borsari 46, 44121 Ferrara, Italy; giuseppe.castaldelli@unife.it (G.C.); luisa.giari@unife.it (L.G.)

**Keywords:** per- and polyfluoroalkyl substances, PFAS, One Health, toxicological pathology, ultrastructural pathology, fish model, translational research

## Abstract

Perfluorooctanoic acid (PFOA), a persistent per- and polyfluoroalkyl substance (PFAS), remains a global toxicological concern due to its ubiquity, bioaccumulation potential, and toxicity even at low concentrations. This study aimed to elucidate the ultrastructural effects of PFOA on the gills of *Cyprinus carpio*, a species of high ecological and trophic relevance. Gill samples from fish experimentally exposed to two PFOA concentrations (200 ng L^−1^ and 2 mg L^−1^), one of which was environmentally relevant, were examined by transmission electron microscopy. The results revealed cytotoxic changes primarily affecting mitochondria-rich (chloride) cells and, to a lesser extent, epithelial and mucous cells. The main alterations included mitochondrial degeneration, Golgi and endoplasmic reticulum stress, and autophagic activation, indicating a coordinated impairment of the endomembrane system. These findings suggest that PFOA induces a bioenergetic and proteo-synthetic imbalance compromising cellular homeostasis. Both direct cytotoxic and indirect endocrine-mediated mechanisms may contribute to the observed lesions. The pronounced sensitivity of mitochondria-rich cells supports their use as generalist biomarkers of PFOA exposure and effect. Within a One Health framework, these cells may also serve as translational models for elucidating conserved subcellular mechanisms of PFAS-induced cytotoxicity across vertebrates, with implications for environmental and human health risk assessment.

## 1. Introduction

Per- and polyfluoroalkyl substances (PFAS), commonly known as “forever chemicals”, comprise a broad group of synthetic organo-fluorine compounds characterised by carbon–fluorine bonds that confer exceptional chemical and thermal stability [1,2,3]. Since their introduction in the 1940s, PFAS have been widely utilised in industrial applications and consumer goods, both as polymers and as performance-enhancing additives [1,2,3].

Initially considered chemically inert and environmentally safe, they were largely overlooked with regard to persistence and toxicity [4]. It is now well established, however, that PFAS are ubiquitous global contaminants, resistant to degradation and capable of accumulation in both environmental matrices and biological systems, including humans [4,5]. Despite growing concern, regulatory frameworks currently cover only a small fraction of the thousands of PFAS in use [6], and critical knowledge gaps remain regarding their health effects and mechanisms of action at environmentally realistic concentrations [4].

Perfluorooctanoic acid (PFOA), one of the most extensively studied PFAS, remains a significant global concern despite the phase-out of production in many industrialised nations, due to its extreme environmental persistence, bioconcentration and bioaccumulation potential, and widespread distribution in aquatic ecosystems [2,3,4,5,7]. The Veneto Region in northern Italy represents one of the largest recorded cases of groundwater PFOA contamination worldwide, affecting several hundred thousand inhabitants and prompting extensive monitoring and health initiatives [8]. These pollution hotspots underscore the enduring public health and environmental relevance of PFOA, particularly in freshwater systems where aquatic organisms and human food chains remain exposed [9,10].

PFOA has been shown to interact with biological systems at multiple levels of organisation, from molecular and cellular to organ and organismal scales, across a broad range of taxa [9,10,11,12,13,14]. Its capacity to bio-magnify, bioconcentrate and bioaccumulate [15,16,17] and interfere with essential cellular functions [18,19,20] underscores the need to evaluate its toxicity through a holistic One Health framework, integrating evidence from environmental, animal, and human health domains.

Of particular concern is the observation that PFOA may induce cellular dysfunction at concentrations below the current analytical limits of detection [21,22,23,24], raising fundamental questions about what constitutes a “safe” exposure threshold. This uncertainty is reflected in the precautionary stance adopted by several regulatory authorities. For instance, the United States Environmental Protection Agency (EPA) has established advisory limits for PFOA in water that lie well below conventional quantification levels, acknowledging both the analytical challenges and the potential for biological effects at ultra-trace concentrations [25,26].

Given the central role of water as the primary vector for the environmental dissemination of PFOA across ecosystems, aquatic organisms, and particularly fish, represent key sentinels for assessing its biological effects [27,28]. As the most widespread group of aquatic vertebrates, fish integrate PFOA exposure through both direct contact with contaminated water and dietary uptake, thereby reflecting its distribution and bioaccumulation within aquatic food webs [27,28]. Consequently, they constitute ideal experimental and monitoring models within both the One Health and translational toxicology frameworks [21,29,30,31,32].

While zebrafish (*Danio rerio*) are widely used in toxicology, their restricted wild distribution limits global representativeness [33,34]. In contrast, common carp (*Cyprinus carpio*), cosmopolitan, ecologically important, and consumed by humans, provide greater environmental and trophic relevance. Their longevity and capacity for bioconcentration and bioaccumulation make them suitable models for assessing persistent contaminants such as PFOA [22,35,36,37].

Morphology has traditionally represented a fundamental approach in the assessment of toxicant-induced effects, PFOA included [22,37,38]. A lesion reflects altered cellular function [22,37,38], and ultrastructural analysis provides the highest resolution for detecting subcellular alterations and visualising fine structural changes underlying functional impairment, thereby linking structure to function in cell pathology [22,37,39,40].

Relatively few studies have investigated structurally or ultra-structurally the PFOA impact on fish [41,42,43,44,45,46], while most previous research has focused on biomolecular, biochemical, omics, or genetic approaches [47,48,49,50,51], leaving morphological analyses relatively underexplored despite their fundamental importance in elucidating pathogenesis [22,37,39,40]. In previous investigations conducted on the same experimental cohort of common carp examined in the present study, PFOA exposure elicited distinct lesions in several organs, including the liver [40]; the kidney, encompassing nephronal, haematopoietic, and thyroid-associated compartments [21,22,23,37,52]; and the gonads [53]. The liver and kidney play central roles in PFOA biotransformation and elimination, respectively, reflecting their metabolic and excretory significance in PFAS toxicokinetics [36,54].

In freshwater fish, which do not actively drink water [36], the gills represent the primary route of PFOA absorption from the aquatic environment, whereas the kidney serves as the major excretory organ [36,54]. Despite this crucial physiological role, the effects of PFOA on the gill structure, and particularly on its ultrastructure, remain poorly characterised, with only limited and often descriptive data currently available [45,46,55]. Given their high metabolic activity and direct interface with the external environment, gills are recognised as one of the most sensitive organs to toxicants and environmental stressors [56,57,58,59,60,61,62,63,64].

The gill interlamellar region constitutes a morphologically dynamic compartment composed of relatively undifferentiated cells as well as fully differentiated, highly specialised cell types, including mucous cells and mitochondria-rich (chloride) cells [65,66,67,68]. This compartment plays a key role in gill remodelling through the coordinated regulation of cell proliferation and apoptosis [65,66,67,68]. Among these, mitochondria-rich cells stand out as particularly distinctive and functionally significant, owing to their involvement in ion transport, osmoregulation and acid-base regulation [69,70,71,72]. Their morphological plasticity and sensitivity to toxicants and variations in environmental parameters make them promising candidates for use as cellular biomarkers of environmental stress and contaminant exposure [58,59,60,73].

Given the physiological importance of gills as both an entry and regulatory site for xenobiotics, and their established sensitivity to chemical and environmental stressors, an in-depth ultrastructural assessment offers a valuable means of elucidating the cellular and subcellular mechanisms underlying PFOA toxicity. In this context, the present study was designed to characterise the ultrastructural alterations induced by sub-chronic exposure to PFOA in the gills of common carp (*C. carpio*), with particular emphasis on mitochondria-rich cells and other specialised branchial cell types. By analysing tissues obtained from a previously established experimental cohort, this work aims to provide new insights into the cellular targets and mechanistic pathways of PFOA-induced cytotoxicity. Accordingly, this work should be regarded as an exploratory, qualitative analysis of PFOA-elicited ultrastructural alterations in carp gills, justified by the paucity of pertinent branchial ultrastructural data, leveraging archival material from a previously established cohort to avoid sacrificing additional fish and to interpret gill findings alongside concurrent lesions characterized by the present research group in other organs and cell types from the same individuals, thereby strengthening evidence for common PFOA-associated pathophysiology. Furthermore, by integrating these findings within a One Health and translational toxicopathological perspective, the study seeks to evaluate the potential of mitochondria-rich cells as biomarkers of exposure and effect, as well as model systems for understanding conserved mechanisms of organelle stress across vertebrates.

## 2. Materials and Methods

The gill tissue ultrathin sections examined in the present study originated from archival material (resin-embedded specimens retained as uncut epoxy resin blocks) obtained during a previously conducted experimental trial [53]. For comprehensive details concerning the experimental design, husbandry conditions, and exposure protocol, readers are directed to that reference [53]. Notably, no additional fish were sacrificed for the purposes of this investigation, in full compliance with ethical and animal welfare principles.

In summary, the material analysed consisted of gill ultrathin sections from fifteen two-year-old common carp from the same parental stock (*C. carpio*; mean total length: 19.3 ± 2.5 cm; mean body mass: 104.8 ± 27.8 g). These samples were drawn from three experimental groups described in the original study [53]. Given the exploratory qualitative ultrastructural endpoints and the hierarchical replication across arches, regions, grids, and sections, a per-group sample size of five individuals per experimental group is consistent with recommended practices for unbiased gill morphology and with prior ultrastructural toxicology studies demonstrating detectable and consistent cellular alterations at similar sampling number [74,75]. Five specimens belonged to the unexposed control group (mean total length 18.6 ± 1.7 cm; mean body mass 100.5 ± 28.9 g), five to a group exposed to 200 ng L^−1^ PFOA (mean total length 21.0 ± 3.4 cm; mean body mass 123.3 ± 25.3 g), and the remaining five to a group exposed to 2 mg L^−1^ PFOA (mean total length 18.4 ± 1.1 cm; mean body mass 92.0 ± 21.2 g). The exposure lasted for 56 days under sub-chronic conditions in a continuous flow-through system [53]. The PFOA concentrations selected were based on their ecological and experimental relevance [53]. The lower concentration (200 ng L^−1^) reflects levels reported in surface waters [76], thereby representing environmentally realistic exposure conditions, while the higher concentration (2 mg L^−1^) was chosen in accordance with previous toxicological studies demonstrating its ability to elicit histopathological changes in cyprinid fish [77]. Gill tissues obtained from the previously conducted experimental exposure [53] were used for ultrastructural examination. In particular, for each fish, two gill arches (the first and the third) were sampled, with two regions per arch (the apical and the middle portion), yielding four samples per fish to ensure anatomical representation.

Samples collected during the previous trial [53] had been processed immediately after euthanasia for transmission electron microscopy following standard procedures. Briefly, the gill fragments were initially fixed in 2.5% glutaraldehyde buffered with sodium cacodylate (pH 7.3) at 4 °C for approximately 3 h, post-fixed in 1% osmium tetroxide for 2 h, dehydrated through a graded acetone series, and subsequently embedded in epoxy resin (Durcupan™ ACM; Fluka, Sigma-Aldrich, St. Louis, MO, USA). All specimens were processed in a single batch using freshly prepared reagents, with standardized fixation/post-fixation times and temperatures applied identically to all experimental groups to minimize potential artefacts; resin-embedded uncut blocks were then sectioned only for the present analysis with a Reichert Om U2 ultramicrotome (Reichert-Jung Co., Heidelburg, Germany). Ultrathin sections (approximately 90 nm) were stained with uranyl acetate and lead citrate and examined using a Talos L120C transmission electron microscope (Thermo Fisher Scientific, Waltham, MA, USA) operated at 120 kV. For each sample, three grids were prepared and examined, and for each grid, approximately ten to twenty ultrathin sections were evaluated. The grids captured different, non-overlapping fields from the same sample; representative micrographs were selected from multiple fields of view across grids and sections to avoid selection bias and to reflect recurring features.

## 3. Results

**Figure 1:**
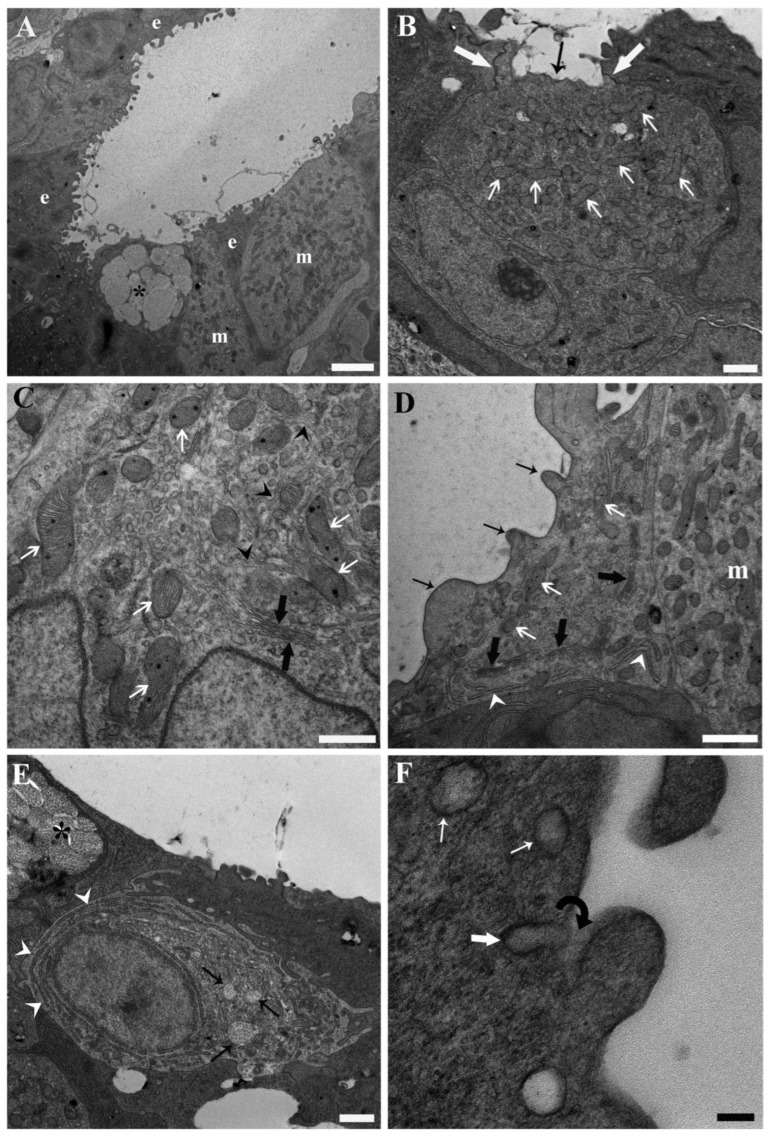
Transmission electron micrographs illustrating the base of the secondary lamellae and the interlamellar space from the gills of unexposed common carp (*Cyprinus carpio*). (**A**) The general tissue architecture at the base of a secondary lamella and within the interlamellar space is shown, highlighting the primary cell types: epithelial cells (e) with distinctive short, squat cytoplasmic projections, mucous cells (asterisk), and large, ovoid mitochondria-rich cells (m) with pale cytoplasm. Scale bar = 2.5 µm. (**B**) A putative β/dark mitochondria-rich cell is depicted, characterised by large mitochondria (white thin arrows) with well-defined cristae and matrix granules, a relatively less prominent tubular system compared to α/light mitochondria-rich cells (see panels (**C**,**D**)), an apical pit (black arrow), and tight junctions (thick white arrows) with adjacent epithelial cells. Scale bar = 1 µm. (**C**) Cytoplasmic detail of a putative α/light mitochondria-rich cell is presented, showing a prominent anastomosing network of tubular structures (tubular system, black arrow heads), a well-developed Golgi apparatus (thick black arrows) with associated vesicles, and abundant voluminous mitochondria (thin white arrows) with evident cristae and matrix granules. Scale bar = 500 nm. (**D**) The cytoplasmic organisation of an epithelial cell is shown, featuring an extensive Golgi apparatus (thick black arrows) with associated vesicles, rough endoplasmic reticulum (white arrow heads), and relatively smaller and less abundant mitochondria (white arrows) compared to the underlying α/light mitochondria-rich cell (m). The short, squat cytoplasmic projections (thin black arrows) are also visible. Scale bar = 1 µm. (**E**) An immature mucous cell is depicted, characterised by abundant rough endoplasmic reticulum (white arrow heads), a well-developed Golgi apparatus with associated vesicles, and developing mucous granules (black arrows). A mature mucous cell (asterisk) lining the epithelial surface is also visible. Scale bar = 1 µm. (**F**) Golgi-derived vesicles (thin white arrows) approaching the epithelial surface are shown, with the fusion of one vesicle (thick white arrow) with the plasma membrane illustrated, suggesting direct continuity between the vesicle contents and the fuzzy coat (black curved arrow) on the outer cell surface. Scale bar = 100 nm.

At the base of the secondary lamellae and within the interlamellar space of unexposed fish, three primary cell types were identified: epithelial cells, mucous cells, and mitochondria-rich cells (Figure 1A). Among these, mitochondria-rich cells were the most prominent specialised cells observed in this region of the branchial epithelium. These cells appeared as large, ovoid structures, characterised by an abundance of substantial mitochondria within their cytoplasm (Figure 1A–D).

Notably, mitochondria-rich cells exhibited a characteristic membranous system comprising the following components: a tubular system (an anastomosing network of tubules) in continuity with the basolateral membrane through membrane infoldings, which enmeshed the mitochondria; endoplasmic reticulum, often interdigitated with the tubular system; a vesiculotubular system, predominantly located in the apical region of the cell and associated with the trans face of the Golgi apparatus (Figure 1C,D).

Referring to the classifications proposed by Pisam [71], most mitochondria-rich cells appeared as α/light cells, characterized by a prominent tubular system indicative of enhanced ion-transport capacity (Figure 1A,C,D). Conversely, darker mitochondria-rich cells, displaying a less extensive tubular system, were occasionally observed and classified as β/dark cells (Figure 1B).

Mucous cells represented another group of specialised cells, displaying their typical ultrastructural features, primarily characterised by the accumulation of mucous granules within their cytoplasm (Figure 1A). Immature mucous cells (Figure 1E) were notable for their abundant rough endoplasmic reticulum, a well-developed Golgi apparatus with associated vesicles, and evidence of developing mucous granules.

Epithelial cells formed the structural scaffold of the branchial epithelium, accommodating the specialised cells mentioned above. These cells were highly pleomorphic, adjusting their morphology to neighbouring cells (Figure 1A,B,D,E). At the ultrastructural level, epithelial cells exhibited large mitochondria, an extensive rough endoplasmic reticulum, and a prominent Golgi complex with numerous associated vesicles (Figure 1D). Frequent fusion of these vesicles with the plasma membrane was observed, suggesting a direct continuity between vesicle contents and the outer cell’s fuzzy coat (Figure 1F).

**Figure 2:**
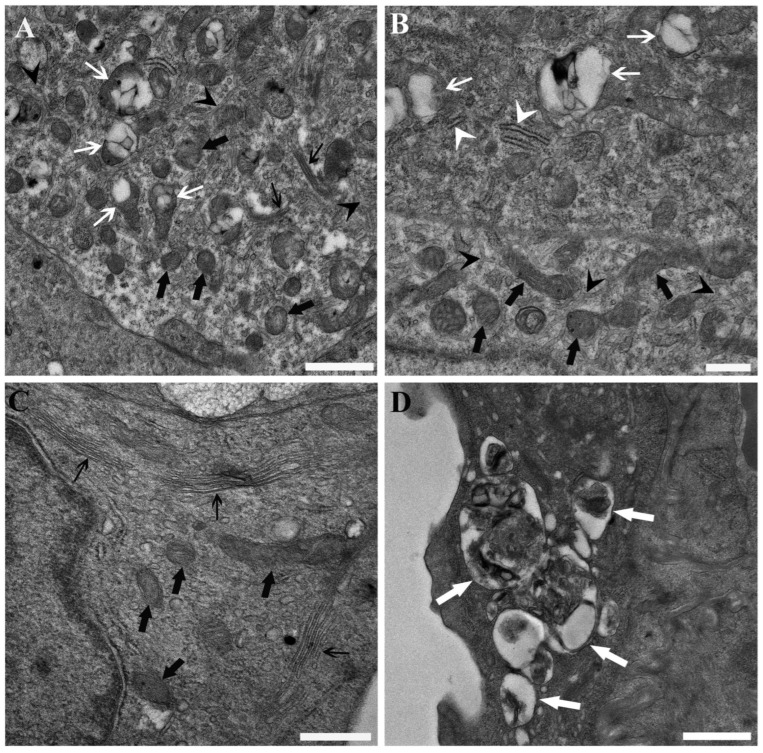
Transmission electron micrographs illustrating the base of the secondary lamellae and the interlamellar space from the gills of common carp (*Cyprinus carpio*) exposed to 200 ng L^−1^ PFOA. (**A**) Mitochondria in a putative α/light mitochondria-rich cell exhibit total or partial ballooning (white arrows), accompanied by cristolysis, matrix dissolution, and the accumulation of electron-dense, membranaceous inclusions. Intact mitochondria (thick black arrows) appear darker, with less distinct cristae and smaller, less numerous matrix granules compared to those documented in unexposed fish. The Golgi apparatus (thin black arrows) is less prominent, with fewer associated vesicles than expected in unexposed fish. The tubular system is also visible (black arrow heads). Scale bar = 1 µm. (**B**) A putative β/dark mitochondria-rich cell (above) and a putative α/light mitochondria-rich cell (below) are shown. In the putative α/light mitochondria-rich cell, mitochondria (thick black arrows) appear dark, with poorly defined cristae and fewer, smaller matrix granules. In contrast, in the putative β/dark mitochondria-rich cell mitochondria (white arrows) are more altered, displaying total or partial ballooning, cristolysis, matrix dissolution, and accumulation of membranaceous inclusions. The tubular system (black arrow heads) and rough endoplasmic reticulum cisternae (white arrow heads) are shown. Scale bar = 500 nm. (**C**) Cytoplasmic details of an epithelial cell reveal a reduction in the size and prominence of the Golgi complex (thin black arrows), with the associated vesicles being more severely affected. Mitochondria (thick black arrows) display diminished matrix granules, less distinct cristae, accompanied by a reduction in the extent of the rough endoplasmic reticulum and the number of associated ribosomes. Scale bar = 500 nm. (**D**) An epithelial cell containing numerous autophagosomes (white arrows) is observed. Scale bar = 1 µm.

PFOA exposure affected the ultrastructural integrity of the cells. At 200 ng L^−1^ PFOA, mitochondria-rich cells exhibited scattered ballooning of mitochondria, accompanied by reduced and smaller matrix granules, less distinct cristae, and a diminished Golgi complex with fewer associated vesicles (Figure 2A). Interestingly, when putative α/light and β/dark cells were observed side by side—a spatial arrangement that enhanced confidence in this direct comparison—the β/dark cells exhibited more pronounced mitochondrial alterations than the α/light cells (Figure 2B). A relative reduction in the size and prominence of the Golgi complex and associated vesicles was also observed in epithelial cells, along with mitochondria displaying reduced and smaller matrix granules and less distinct cristae. Furthermore, the rough endoplasmic reticulum appeared compromised, showing reductions in both its extent and the number of associated ribosomes (Figure 2C). In some instances, evidence of autophagocytosis was also identified (Figure 2D).

**Figure 3:**
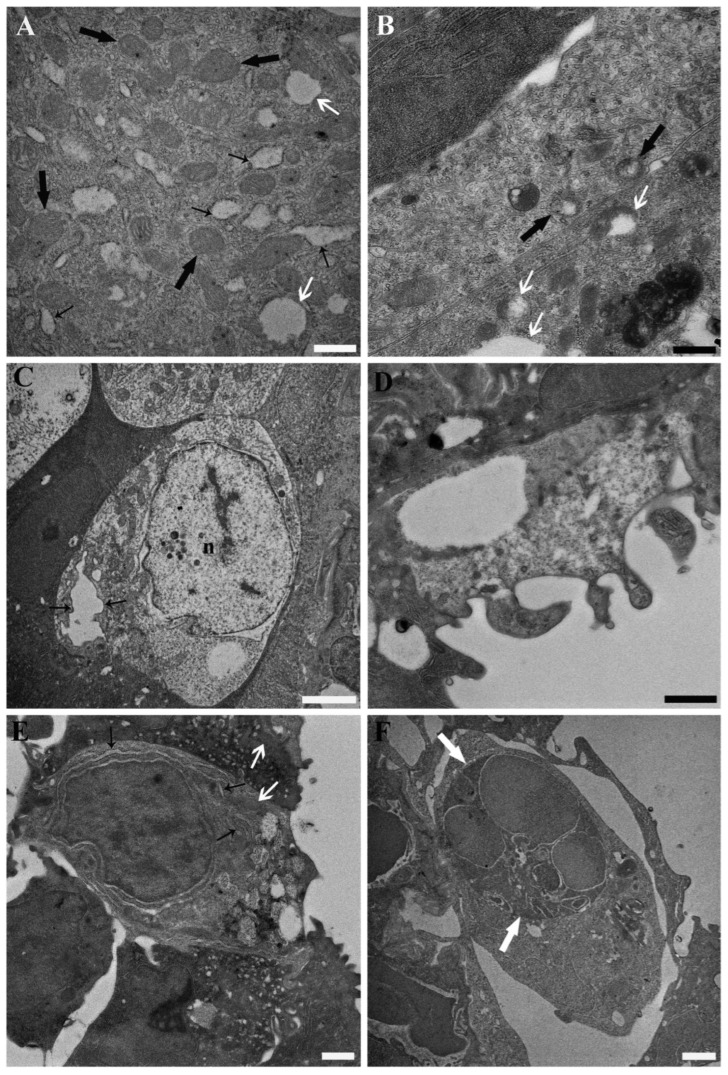
Transmission electron micrographs illustrating the base of the secondary lamellae and the interlamellar space from the gills of common carp (*Cyprinus carpio*) exposed to 2 mg L^−1^ PFOA. (**A**) A putative α/light mitochondria-rich cell shows mitochondrial ballooning (white arrows) and enlargement of the cisternae of the rough endoplasmic reticulum (thin black arrows), with accumulation of flocculent material inside. In general, mitochondria (thick black arrows) appear dark, with poorly defined cristae and fewer, smaller matrix granules. Scale bar = 500 nm. (**B**) A putative α/light mitochondria-rich cell (above) displays dark mitochondria with poorly defined cristae, fewer and smaller matrix granules, and incipient matrix dissolution or ballooning (black arrows). Conversely, mitochondria of a putative β/dark mitochondria-rich cell (below) show more pronounced alterations, including total or partial ballooning (white arrows). Scale bar = 500 nm. (**C**) A necrotic putative α/light mitochondria-rich cell is shown, characterised by a karyolitic nucleus (n) and an enlarged endoplasmic reticulum (arrows). Scale bar = 2 µm. (**D**) A necrotic epithelial cell shows total disarrangement of the cytoplasmic architecture. Scale bar = 1 µm. (**E**) In a mucous cell, mitochondria (white arrows) are difficult to discern, appearing very dark and lacking evident cristae. Enlargement and degranulation of the cisternae of the rough endoplasmic reticulum are also evident (black arrows). Notably, no Golgi apparatus or associated vesicles are discernible. Scale bar = 1 µm. (**F**) A phagocyte with a large phagosome (white arrows) is observed beneath the epithelial lining at the base of a secondary lamella. Scale bar = 1 µm.

At the highest tested concentration of PFOA (2 mg L^−1^), the previously reported ultrastructural alterations were exacerbated, particularly in mitochondria-rich cells. These alterations included mitochondrial ballooning, enlargement of the cisternae of the rough endoplasmic reticulum, and the apparent disappearance of the Golgi complex and associated vesicles (Figure 3A,B). Evidence of necrosis was also observed in individual mitochondria-rich cells and epithelial cells (Figure 3C,D). Mucous cells appeared less affected by PFOA exposure compared to other cell types. However, at the highest tested concentration, mitochondrial alterations were consistently evident in all exposed individuals, including matrix dissolution, darkening, and reduced visibility of cristae. These were accompanied by somewhat enlarged rough endoplasmic reticulum cisternae and reduced or absent Golgi complex and associated vesicles (Figure 3E). Additionally, recruitment of granulocytes and macrophages was observed (Figure 3F).

The described ultrastructural alterations were reproducible across exposed fish and not observed in unexposed control fish. Given the strictly standardized, batch-processed preparation, these findings are unlikely to reflect fixation or processing artefacts and are instead consistent with PFOA-associated cytotoxic changes.

## 4. Discussion

The architecture of the gill interlamellar space documented in the present study aligns with previous descriptions in the literature, supporting its characterisation as a plastic and dynamic anatomical site. This space accommodates relatively undifferentiated cells alongside fully differentiated, highly specialised cells, such as mucous cells and mitochondria-rich cells, and plays a role in gill remodelling through the balance between cell proliferation and cell death [65,66,67,68]. In particular, within the multilayered interlamellar filamental epithelium, pavement and, in some instances, columnar epithelial cells can be observed lining the surface and exhibiting characteristic micro-ridges that somewhat resemble fingerprints [78,79]. A prominent Golgi apparatus, an extensive endoplasmic reticulum, and vesicles opening at the external surface have previously been described in these cells, suggesting a secretory function [68,78,80]. These pavement cells typically display low mitochondrial densities and, in some species, possess apical secretory granules, characteristics consistent with their established morphology in classical ultrastructural surveys [68]. This contrasts with the squamous epithelial cells lining the lamellar edges, in which these secretory features have not been reported [68,78,80].

Mitochondria-rich cells (also referred to as chloride cells) represent the most distinctive cellular type within the gill epithelium and are also found in other water-lining epithelia, particularly during the larval stage [81]. The number and morphology of mitochondria-rich cells vary among fish species and according to the aquatic environment. Moreover, distinct subtypes have been described, displaying specific ultrastructural characteristics and potentially differing functional roles [71,82,83].

The present study demonstrated that PFOA exposure induced alterations in the ultrastructural organisation of the three principal branchial cell types examined, with the severity of these changes increasing according to PFOA dose. The results should be considered from a One Health perspective, as they have implications not only for fish themselves, both wild and farmed, but also for their role as a food source in animal and human diets. Moreover, these findings can be interpreted within the framework of translational research. Mitochondria-rich cells are not unique to fish, being also present in amphibians, reptiles, and birds [84,85]; however, they have not been reported in mammals, which appear to lack direct structural analogues, although cells with comparable ultrastructural features and/or functions are certainly present. For instance, structural and cytochemical similarities have been described between mammalian tuft cells and chloride (mitochondria-rich) cells [86]. Furthermore, from an osmoregulatory perspective, certain epithelia of other vertebrates, such as the frog skin, the urinary bladder epithelium of toads and turtles, and the epithelium of the cortical collecting duct in mammalian kidneys, share several morphological and functional characteristics with the gill epithelium of freshwater fish [87].

Given their primary role in osmoregulation, mitochondria-rich cells may thus serve as a valuable model for investigating and interpreting the effects of toxicants, including PFOA, on conserved cellular structures and functions. Very interestingly, in the same carp examined in the present experimental set, focal vesiculation and spotted matrix rarefaction of mitochondria were observed in the main/clear cells of the nephron collecting ducts. These mitochondrial alterations were more pronounced in the collecting ducts compared with other tubular segments. This finding led to speculation regarding the possible presence and role of organic anion transporters (OATs) in the carp renal collecting duct [23], analogous to those described in mammals [88], in which per- and polyfluoroalkyl substances (PFAS) are known to be excreted via OAT-mediated pathways [89].

As a possible research follow-up, it should be investigated whether PFOA reaches the target cells predominantly through absorption from the surrounding water or through secretion via the bloodstream. It must be considered that PFOA is absorbed primarily at the external interface represented by the gills [36], since freshwater fish do not actively drink water [36] and the kidney represents the main route of excretion [54]. Interestingly, xenobiotic transporter activity has been described in ionocytes (namely mitochondria-rich cells) of zebrafish embryos, suggesting a possible protective role of these cells in xenobiotics excretion and highlighting potential functional similarities with mammalian renal cells [90]. In other words, although the gills constitute the main absorption site and the kidney the principal excretion route, mitochondria-rich cells may locally accumulate higher PFOA concentrations as a consequence of a xenobiotic transporter-mediated process, with PFOA originating from the blood, the tissue compartment known to exhibit the highest PFOA concentration [36,54], as confirmed in a previous study on the same carp used in the present experimental set [53]. This process is likely negligible from a toxicokinetic perspective, in terms of its overall contribution to excretion [36], but may nonetheless be sufficient to account for the greater ultrastructural alterations observed in mitochondria-rich cells compared with other cell types. The observation that β/dark cells, which have been reported to possess a distinct association with the gill central venous sinus [71], exhibited more severe ultrastructural alterations supports the hypothesis of their greater exposure to circulating PFOA. Notably, the gill central venous sinus is part of the so-called secondary vascular system in teleosts, which drains a portion of the blood from the efferent branchial artery [91]. This drained blood returns directly to the heart, partially bypassing the systemic circulation and establishing a localised recirculation of branchial blood [91]. Such a “branchial–cardiac circulation” may influence the local distribution and persistence of blood-borne xenobiotics, such as PFOA, at the gill level. However, this potential higher exposure should be regarded at the cellular rather than the tissue level, since gills do not exhibit either particularly high PFOA concentrations at equilibrium or a significant role in PFOA excretion [36]. Rather, localised accumulation within mitochondria-rich cells, particularly β/dark subtypes, may result from their close vascular association and possible xenobiotic transporter activity, leading to greater ultrastructural vulnerability. Interestingly, a comparable vascular mechanism has previously been discussed in relation to the response of another teleost-specific cell type, the rodlet cell, in fish experimentally exposed to a toxicant [92]. The analogous, albeit less severe, ultrastructural alterations observed in the other cell types should be interpreted within a similar mechanistic framework, namely as a consequence of PFOA uptake from interstitial fluids and, ultimately, from the bloodstream. This interpretation is indirectly supported by the observation that, within the present study, only polygonal epithelial cells, known for their secretory activity [68,78,80] and, consequently, for their greater metabolic dependence on blood-borne nutrient supply, displayed discernible ultrastructural alterations. Such dependence implies a more sustained contact with circulating PFOA. Conversely, the more flattened squamous epithelial cells, despite their closer anatomical proximity to the external environment and thus to the relatively lower PFOA concentrations dissolved in water, exhibited minimal or no detectable alterations following exposure. This differential cellular response supports the view that blood-mediated PFOA delivery, rather than direct branchial absorption from water, represents the predominant route influencing the observed ultrastructural changes. It should be emphasised, however, that in a study on adult euryhaline *Oryzias melastigma*, Avellán-Llaguno et al. (2020) [93] demonstrated that elevated salinity upregulated the expression of organic anion transporter 1 (OAT1) and fatty acid-binding protein (FABP) in gill tissues, thereby facilitating the uptake of PFOA and other perfluoroalkyl acids. This apparent contradiction with findings from zebrafish embryos, in which mitochondria-rich cells exhibit xenobiotic transporter activity primarily associated with excretion [90], likely reflects fundamental differences in species, developmental stage, and environmental salinity (saltwater versus freshwater). In adult euryhaline teleosts, gill epithelia function as dynamic osmoregulatory interfaces, where salinity-dependent modulation of carrier proteins may promote bidirectional, or even net inward, transport of amphiphilic anions such as PFOA [93]. Conversely, in freshwater or embryonic models, where osmotic gradients are minimal and renal excretory pathways are immature, OATs may predominantly act to limit xenobiotic accumulation [90]. Nevertheless, targeted studies on adult common carp should be undertaken to directly test this hypothesis through OAT1 immunohistochemistry to localize transporter expression in specific branchial cell types, combined with transporter-inhibition assays and targeted transcriptomic profiling to clarify the possible role of such carriers in the influx–efflux balance and to determine whether specific gill cell types preferentially contribute to PFOA concentration and retention.

As previously noted, mitochondria were the most affected organelles. PFOA exposure is well documented to impair mitochondrial structure and function [50], and comparable alterations have been reported in the liver, kidney, and, to a lesser extent, in the thyroid follicular epithelium of the same carp used in the present experimental set [22,23,40]. Regarding the mechanistic basis of these alterations, it has been proposed that PFOA, owing to its surfactant properties, disrupts mitochondrial integrity by altering both the inner and outer membranes. Such disruption compromises the electron transport chain, leading to the excessive generation of reactive oxygen species (ROS) [50]. Additionally, modulation of the mitochondrial permeability transition pore has been suggested as a contributing mechanism [94,95]. Furthermore, given PFOA’s structural similarity to fatty acids and its ability to modify mitochondrial membrane fluidity, it may interfere with β-oxidation of fatty acids and/or uncouple oxidative phosphorylation [40]. Collectively, these mechanisms could converge to explain the profound mitochondrial damage observed ultra-structurally, which likely represents a central event in PFOA-induced cytotoxicity.

With regard to the Golgi apparatus, its loss has been reported in the liver of mice orally administered PFOA. In particular, Golgin-97, a membrane protein of the trans-Golgi network, was inhibited even at the lowest tested PFOA concentration [96]. The involvement of Golgi stress has also been hypothesised [96], whereby disruption of the mechanisms maintaining Golgi integrity activates a Golgi-dedicated degradation signalling pathway. This, in turn, may trigger autophagy as part of an organelle self-regulatory or protective response [97]. The present findings, which revealed a reduction in the size and prominence of the Golgi complex and its associated vesicles in branchial mitochondria-rich, epithelial, and mucous cells at the highest tested PFOA concentration, may be interpreted within the same mechanistic framework. In this context, PFOA-induced Golgi stress likely occurs in these cells, initiating degradative or autophagic responses aimed at restoring organellar and cellular homeostasis [97].

The ultrastructural evidence of altered rough endoplasmic reticulum (ER) observed in the present study, characterised by dilation of cisternae and ribosomal detachment, should also be taken into account, as it further supports the hypothesis of organelle stress. ER stress has already been proposed as a key mechanism in PFOA-induced hepatotoxicity in carp [98], as well as in other animal models [99,100]. Given the functional continuity between the ER and Golgi apparatus, stress originating in one compartment is likely to propagate to the other, establishing a self-perpetuating cycle of organelle dysfunction. Interestingly, an endoplasmic reticulum–mitochondria communication pathway has been demonstrated in vitro in hepatocytes, where PFOA-induced ER stress triggered mitochondrial-mediated apoptosis [100]. This highlights the complex interplay among organelles in maintaining cellular homeostasis, whereby stress originating in the ER or Golgi can affect mitochondria, while conversely, mitochondrial dysfunction may impair ER and Golgi integrity, generating a coordinated cascade of organelle dysfunction that ultimately compromises cell viability [97].

Collectively, the concurrent alterations observed in mitochondria, the endoplasmic reticulum, the Golgi apparatus, and autophagic activity in the present study point to a coordinated impairment of the endomembrane system as a central event in the concentration-dependent cytotoxic effects of PFOA on branchial cells.

It should be emphasised that the reported ultrastructural alterations may result from both direct cytotoxic effects, previously discussed, and indirect endocrine-mediated mechanisms of PFOA action, which are not mutually exclusive. In the same fish examined in the present study, thyroid alterations consistent with hypothyroidism were previously reported [21,22]. Given that thyroid hormones are known to modulate osmoregulatory function in euryhaline species, stimulating the proliferation and hypertrophy of mitochondria-rich (chloride) cells [101], it is plausible that PFOA-induced thyroid dysfunction may have secondarily contributed to the branchial alterations observed. To achieve a more interpretative understanding of these findings, endocrine function should be systematically assessed in future studies. Moreover, other endocrine pathways, particularly those involving cortisol, should be considered given the established bidirectional interaction between the thyroid and inter-renal axes in teleosts. This interplay is crucial for coordinating osmoregulatory and metabolic processes, as thyroid hormones modulate cortisol-mediated stress responses while cortisol, in turn, influences thyroidal activity, ensuring the integrated regulation of ionic and energetic homeostasis under environmental challenge [102]. Further insights into the potential endocrine-mediated influence of PFOA on branchial physiology were provided by Lu et al. (2021) [45], who reported alterations in gill morphology, ion content, Na^+^/K^+^-ATPase activity, and the transcription of ion transporters and hormone receptor genes in *Oryzias melastigma* following exposure. However, their observations were based solely on light microscopy of paraffin-embedded sections, which is inadequate to resolve the fine architecture of the gill epithelium or to evaluate mitochondria-rich cells, the key effectors of ionic regulation. Although direct cytotoxic effects were not investigated, their molecular data suggest that PFOA can interfere with osmoregulatory and endocrine-related pathways, consistent with a possible hormonal modulation of branchial function [45]. These findings therefore complement the present ultrastructural evidence by indicating that PFOA may act through both cellular (cytotoxic) and endocrine-mediated processes. Recent single-cell transcriptomic evidence further supports the hypothesis that PFOA directly targets ionocytes (mitochondria-rich cells). Yu et al. (2022) [103] identified significant alterations in the expression of key osmoregulatory and structural genes, including *atp1a1a.4* (encoding the Na^+^/K^+^-ATPase α-subunit) and *col1a1a* (encoding the collagen type I α1 chain), in zebrafish embryos exposed to sublethal concentrations of PFOA. Such transcriptional perturbations suggest impairment of ion transport processes and epithelial structural integrity, consistent with the ultrastructural alterations observed in the present study and further reinforce the view that ionocytes (mitochondria-rich cells) represent primary cellular targets in PFOA-induced disruption of osmoregulatory and bioenergetic homeostasis.

Notwithstanding its exploratory and qualitative nature, the present study provides essential descriptive baseline data on PFOA-induced gill ultrastructure that, to the authors’ knowledge, represents the first comprehensive ultrastructural characterization of PFOA effects on fish branchial tissues. Such foundational observations hold intrinsic scientific merit for understanding cellular responses to this persistent contaminant. Nevertheless, these findings underscore the need for future targeted semi-quantitative scoring or quantitative morphometric studies to quantify the magnitude and cellular heterogeneity of ultrastructural alterations, permit rigorous statistical comparison across exposure scenarios, and establish the mechanistic and biomarker applications of the present discoveries.

## 5. Conclusions

This study provides ultrastructural evidence that PFOA induces concentration-dependent cytotoxic effects in the gills of *C. carpio*, primarily affecting mitochondria-rich cells and, to a lesser extent, epithelial and mucous cells. The concurrent alterations detected in mitochondria, the Golgi apparatus, the endoplasmic reticulum, and autophagic activity indicate a coordinated disruption of the endomembrane system as a central pathogenic mechanism. Such organelle impairment reflects a broader bioenergetic and proteo-synthetic imbalance, ultimately leading to the loss of cellular homeostasis. These findings highlight mitochondrial and endomembrane stress as pivotal components of PFOA toxicity and suggest that indirect endocrine-mediated effects may co-occur with direct cytotoxic mechanisms, contributing to the overall pathophysiological response.

From a One Health perspective, the present results hold significance for environmental, animal, and human health, given the ecological and trophic importance of fish species. The pronounced susceptibility of mitochondria-rich cells supports their application as generalist biomarkers of PFOA exposure and effect. Furthermore, owing to their conserved structural and functional analogies with mammalian epithelial cells involved in ion transport and detoxification, mitochondria-rich cells may serve as valuable translational models for elucidating conserved cellular mechanisms of organelle stress and xenobiotic-induced injury. Consequently, these cells can be regarded as reliable biomarkers of One Health relevance and translational significance.

To consolidate and extend these findings, targeted semi-quantitative scoring or quantitative morphometric studies are necessary to confirm the magnitude and cellular heterogeneity of PFOA-induced ultrastructural alterations, elucidate their mechanistic basis, and establish their translational and biomarker applications.

Future research should integrate targeted biomolecular approaches to delineate the molecular cascades underlying organelle dysfunction and to identify the signalling networks connecting mitochondrial, endoplasmic reticulum, and Golgi stress responses. In particular, investigations into the crosstalk between bioenergetic pathways and protein synthesis regulation under PFOA exposure may provide critical insights into the mechanisms linking subcellular damage to systemic endocrine and physiological outcomes.

## Data Availability

All relevant data is reported in the manuscript.

## References

[1] Leung S.C.E., Wanninayake D., Chen D., Nguyen N.-T., Li Q. (2023). Physicochemical Properties and Interactions of Perfluoroalkyl Substances (PFAS)—Challenges and Opportunities in Sensing and Remediation. Sci. Total Environ..

[2] Ding G., Peijnenburg W.J.G.M. (2013). Physicochemical Properties and Aquatic Toxicity of Poly- and Perfluorinated Compounds. Crit. Rev. Environ. Sci. Technol..

[3] Cousins I.T., Dewitt J.C., Glüge J., Goldenman G., Herzke D., Lohmann R., Miller M., Ng C.A., Scheringer M., Vierke L. (2020). Strategies for Grouping Per-and Polyfluoroalkyl Substances (PFAS) to Protect Human and Environmental Health. Environ. Sci. Process. Impacts.

[4] Sinclair G.M., Long S.M., Jones O.A.H. (2020). What Are the Effects of PFAS Exposure at Environmentally Relevant Concentrations?. Chemosphere.

[5] Evich M.G., Davis M.J.B., McCord J.P., Acrey B., Awkerman J.A., Knappe D.R.U., Lindstrom A.B., Speth T.F., Tebes-Stevens C., Strynar M.J. (2022). Per- and Polyfluoroalkyl Substances in the Environment. Science.

[6] Yu R.-S., Yu H.-C., Yang Y.-F., Singh S. (2025). A Global Overview of Per- and Polyfluoroalkyl Substance Regulatory Strategies and Their Environmental Impact. Toxics.

[7] Vierke L., Staude C., Biegel-Engler A., Drost W., Schulte C. (2012). Perfluorooctanoic Acid (PFOA)—Main Concerns and Regulatory Developments in Europe from an Environmental Point of View. Environ. Sci. Eur..

[8] Pivato A., Beggio G., Maggi S., Marrone F., Bonato T., Peres F., Peng W., Lavagnolo M.C. (2024). The Presence of PFAS in Wastes and Related Implications on the Current and Proposed European Regulatory Framework: A Systemic Critical Review. Detritus.

[9] Wasel O., Thompson K.M., Freeman J.L. (2022). Assessment of Unique Behavioral, Morphological, and Molecular Alterations in the Comparative Developmental Toxicity Profiles of PFOA, PFHxA, and PFBA Using the Zebrafish Model System. Environ. Int..

[10] Hayman N.T., Rosen G., Colvin M.A., Conder J., Arblaster J.A. (2021). Aquatic Toxicity Evaluations of PFOS and PFOA for Five Standard Marine Endpoints. Chemosphere.

[11] Teaf C.M., Garber M.M., Covert D.J., Tuovila B.J. (2019). Perfluorooctanoic Acid (PFOA): Environmental Sources, Chemistry, Toxicology, and Potential Risks. Soil Sediment Contam. Int. J..

[12] Huang H., Lyu X., Xiao F., Fu J., Xu H., Wu J., Sun Y. (2024). Three-Year Field Study on the Temporal Response of Soil Microbial Communities and Functions to PFOA Exposure. J. Hazard. Mater..

[13] Hanson M.L., Small J., Sibley P.K., Boudreau T.M., Brain R.A., Mabury S.A., Solomon K.R. (2005). Microcosm Evaluation of the Fate, Toxicity, and Risk to Aquatic Macrophytes from Perfluorooctanoic Acid (PFOA). Arch. Environ. Contam. Toxicol..

[14] Li Y., Yao J., Pan Y., Dai J., Tang J. (2023). Trophic Behaviors of PFOA and Its Alternatives Perfluoroalkyl Ether Carboxylic Acids (PFECAs) in a Coastal Food Web. J. Hazard. Mater..

[15] Boisvert G., Sonne C., Rigét F.F., Dietz R., Letcher R.J. (2019). Bioaccumulation and Biomagnification of Perfluoroalkyl Acids and Precursors in East Greenland Polar Bears and Their Ringed Seal Prey. Environ. Pollut..

[16] Simonnet-Laprade C., Budzinski H., Maciejewski K., Menach K.L., Santos R., Alliot F., Goutte A., Labadie P. (2019). Biomagnification of Perfluoroalkyl Acids (PFAAs) in the Food Web of an Urban River: Assessment of the Trophic Transfer of Targeted and Unknown Precursors and Implications. Environ. Sci. Process. Impacts.

[17] Cheng H., Lv C., Li J., Wu D., Zhan X., Song Y., Zhao N., Jin H. (2022). Bioaccumulation and Biomagnification of Emerging Poly- and Perfluoroalkyl Substances in Marine Organisms. Sci. Total Environ..

[18] Qi Z., Yang X., Geng Z., Zhang L., Huang Z., Shi C., Xie Q., Zhang F. (2025). Health Effects of PFASs on Five Major Human Cancers: A Network Toxicology Perspective on Molecular Pathogenesis. J. Environ. Sci..

[19] Li K., Gao P., Xiang P., Zhang X., Cui X., Ma L.Q. (2017). Molecular Mechanisms of PFOA-Induced Toxicity in Animals and Humans: Implications for Health Risks. Environ. Int..

[20] Zhou J.-X., Qin X.-D., Liu X., He W.-T., Zeeshan M., Dharmage S.C., Perret J., Bui D., Zhang Y.-T., Sun M.-K. (2025). Exposure-Effect of PFOS and PFOA on Lung Function: An Integrated Approach with Epidemiological, Cellular, and Animal Studies. Environ. Res..

[21] Manera M., Giari L. (2024). Segmentation of Renal Thyroid Follicle Colloid in Common Carp: Insights into Perfluorooctanoic Acid-Induced Morphometric Alterations. Toxics.

[22] Manera M., Castaldelli G., Giari L. (2022). Perfluorooctanoic Acid Affects Thyroid Follicles in Common Carp (*Cyprinus carpio*). Int. J. Environ. Res. Public Health.

[23] Manera M., Castaldelli G., Guerranti C., Giari L. (2022). Effect of Waterborne Exposure to Perfluorooctanoic Acid on Nephron and Renal Hemopoietic Tissue of Common Carp *Cyprinus carpio*. Ecotoxicol. Environ. Saf..

[24] Rotondo J.C., Giari L., Guerranti C., Tognon M., Castaldelli G., Fano E.A., Martini F. (2018). Environmental Doses of Perfluorooctanoic Acid Change the Expression of Genes in Target Tissues of Common Carp. Environ. Toxicol. Chem..

[25] Health and Ecological Criteria Division—Office of Science and Technology—Office of Water (2022). Interim Drinking Water Health Advisory: Perfluorooctanoic Acid (PFOA) CASRN 335-67-1.

[26] Rehman A.U., Crimi M., Andreescu S. (2023). Current and Emerging Analytical Techniques for the Determination of PFAS in Environmental Samples. Trends Environ. Anal. Chem..

[27] Lee J.W., Choi K., Park K., Sung C., Yu S.D., Kim P., Seong C., Yu S.D., Kim P. (2020). Adverse Effects of Perfluoroalkyl Acids on Fish and Other Aquatic Organisms: A Review. Sci. Total Environ..

[28] Du D., Lu Y., Zhou Y., Li Q., Zhang M., Han G., Cui H., Jeppesen E. (2021). Bioaccumulation, Trophic Transfer and Biomagnification of Perfluoroalkyl Acids (PFAAs) in the Marine Food Web of the South China Sea. J. Hazard. Mater..

[29] Ma T., Wu P., Wang L., Li Q., Li X., Luo Y. (2023). Toxicity of Per- and Polyfluoroalkyl Substances to Aquatic Vertebrates. Front. Environ. Sci..

[30] Friese C., Nuyts N. (2017). Posthumanist Critique and Human Health: How Nonhumans (Could) Figure in Public Health Research. Crit. Public Health.

[31] Haschek W.M., Rousseaux C.G., Wallig M.A. (2013). Toxicologic Pathology: An Introduction. Haschek and Rousseaux’s Handbook of Toxicologic Pathology, Third Edition: Volume 1–3.

[32] Manera M. (2024). Rodlet Cell Morpho–Numerical Alterations as Key Biomarkers of Fish Responses to Toxicants and Environmental Stressors. Toxics.

[33] Choi T.-Y., Choi T.-I., Lee Y.-R., Choe S.-K., Kim C.-H. (2021). Zebrafish as an Animal Model for Biomedical Research. Exp. Mol. Med..

[34] Manera M. (2024). Texture Analysis as a Discriminating Tool: Unmasking Rodlet Cell Degranulation in Response to a Contaminant of Emerging Concern. Front. Biosci.-Landmark.

[35] Kim W.-K., Lee S.-K., Jung J. (2010). Integrated Assessment of Biomarker Responses in Common Carp (*Cyprinus carpio*) Exposed to Perfluorinated Organic Compounds. J. Hazard. Mater..

[36] Petre V.A., Chiriac F.L., Lucaciu I.E., Paun I., Pirvu F., Iancu V.I., Novac L., Gheorghe S. (2023). Tissue Bioconcentration Pattern and Biotransformation of Per-Fluorooctanoic Acid (PFOA) in *Cyprinus carpio* (European Carp)—An Extensive In Vivo Study. Foods.

[37] Manera M., Casciano F., Giari L. (2023). Ultrastructural Alterations of the Glomerular Filtration Barrier in Fish Experimentally Exposed to Perfluorooctanoic Acid. Int. J. Environ. Res. Public Health.

[38] Wallig M.A., Janovitz E.B., Wallig M.A., Haschek W.M., Rousseaux C.G., Bolon B. (2018). Morphologic Manifestations of Toxic Cell Injury. Fundamentals of Toxicologic Pathology.

[39] Grover A., Sinha R., Jyoti D., Faggio C. (2021). Imperative Role of Electron Microscopy in Toxicity Assessment: A Review. Microsc. Res. Tech..

[40] Manera M., Castaldelli G., Fano E.A., Giari L. (2021). Perfluorooctanoic Acid-Induced Cellular and Subcellular Alterations in Fish Hepatocytes. Environ. Toxicol. Pharmacol..

[41] Wu D.-L., Cheng L., Rao Q.-X., Wang X.-L., Zhang Q.-C., Yao C.-X., Chen S.-S., Liu X., Song W., Zhou J.-X. (2022). Toxic Effects and Transcriptional Responses in Zebrafish Liver Cells Following Perfluorooctanoic Acid Exposure. Aquat. Toxicol..

[42] Han P., Xue Y., Sun Z., Liu X., Miao L., Yuan M., Wang X. (2025). The Toxicological Effects of Perfluorooctanoic Acid (PFOA) Exposure in Large Yellow Croaker (*Larimichthys crocea*): Exploring the Relationship between Liver Damage and Gut Microbiota Dysbiosis. Environ. Res..

[43] Lee J.W.J.W., Lee J.W.J.W., Kim K., Shin Y.J., Kim J., Kim S., Kim H., Kim P., Park K. (2017). PFOA-Induced Metabolism Disturbance and Multi-Generational Reproductive Toxicity in *Oryzias latipes*. J. Hazard. Mater..

[44] Zhan M., Shi K., Zhang X., Fan Q., Xu Q., Liu X., Li Z., Liu H., Xia Y., Sha Z. (2023). Histological Assessment and Transcriptome Analysis Provide Insights into the Toxic Effects of Perfluorooctanoic Acid to Juvenile Half Smooth Tongue Sole *Cynoglossus semilaevis*. J. Ocean Univ. China.

[45] Lu W., Long L., Zhao P., Zhang X., Yan C., Dong S., Huang Q. (2021). Perfluorinated Compounds Disrupted Osmoregulation in *Oryzias melastigma* during Acclimation to Hypoosmotic Environment. Ecotoxicol. Environ. Saf..

[46] Liu Y., Wang J., Wei Y., Zhang H., Liu Y., Dai J. (2008). Molecular Characterization of Cytochrome P450 1A and 3A and the Effects of Perfluorooctanoic Acid on Their mRNA Levels in Rare Minnow (*Gobiocypris rarus*) Gills. Aquat. Toxicol..

[47] Jantzen C.E., Toor F., Annunziato K.A., Cooper K.R. (2017). Effects of Chronic Perfluorooctanoic Acid (PFOA) at Low Concentration on Morphometrics, Gene Expression, and Fecundity in Zebrafish (*Danio rerio*). Reprod. Toxicol..

[48] Jantzen C.E., Annunziato K.M., Cooper K.R. (2016). Behavioral, Morphometric, and Gene Expression Effects in Adult Zebrafish (*Danio rerio*) Embryonically Exposed to PFOA, PFOS, and PFNA. Aquat. Toxicol..

[49] Han Z., Liu Y., Wu D., Zhu Z., Lü C. (2012). Immunotoxicity and Hepatotoxicity of PFOS and PFOA in Tilapia (*Oreochromis niloticus*). Chin. J. Geochem..

[50] Hagenaars A., Vergauwen L., Benoot D., Laukens K., Knapen D. (2013). Mechanistic Toxicity Study of Perfluorooctanoic Acid in Zebrafish Suggests Mitochondrial Dysfunction to Play a Key Role in PFOA Toxicity. Chemosphere.

[51] Miranda A.F., Trestrail C., Lekamge S., Nugegoda D. (2020). Effects of Perfluorooctanoic Acid (PFOA) on the Thyroid Status, Vitellogenin, and Oxidant–Antioxidant Balance in the Murray River Rainbowfish. Ecotoxicology.

[52] Manera M., Castaldelli G., Giari L. (2023). Perfluorooctanoic Acid Promotes Recruitment and Exocytosis of Rodlet Cells in the Renal Hematopoietic Tissue of Common Carp. Toxics.

[53] Giari L., Vincenzi F., Badini S., Guerranti C., Dezfuli B.S., Fano E.A., Castaldelli G. (2016). Common Carp *Cyprinus carpio* Responses to Sub-Chronic Exposure to Perfluorooctanoic Acid. Environ. Sci. Pollut. Res..

[54] Consoer D.M., Hoffman A.D., Fitzsimmons P.N., Kosian P.A., Nichols J.W. (2014). Toxicokinetics of Perfluorooctanoate (PFOA) in Rainbow Trout (*Oncorhynchus mykiss*). Aquat. Toxicol..

[55] Coy C.O., Steele A.N., Abdulelah S.A., Belanger R.M., Crile K.G., Stevenson L.M., Moore P.A. (2022). Differing Behavioral Changes in Crayfish and Bluegill under Short- and Long-Chain PFAS Exposures: Field Study in Northern Michigan, USA. Ecotoxicol. Environ. Saf..

[56] Manera M., Sayyaf Dezfuli B., DePasquale J.A., Giari L. (2016). Multivariate Approach to Gill Pathology in European Sea Bass after Experimental Exposure to Cadmium and Terbuthylazine. Ecotoxicol. Environ. Saf..

[57] Manera M., Giari L., De Pasquale J.A., Sayyaf Dezfuli B. (2016). Local Connected Fractal Dimension Analysis in Gill of Fish Experimentally Exposed to Toxicants. Aquat. Toxicol..

[58] Manera M., Giari L., DePasquale J.A., Dezfuli B.S.S. (2016). European Sea Bass Gill Pathology after Exposure to Cadmium and Terbuthylazine: Expert versus Fractal Analysis. J. Microsc..

[59] Giari L., Manera M., Simoni E., Dezfuli B.S.S. (2006). Changes to Chloride and Rodlet Cells in Gills, Kidney and Intestine of *Dicentrarchus labrax* (L.) Exposed to Reduced Salinities. J. Fish Biol..

[60] Pawert M., Müller E., Triebskorn R. (1998). Ultrastructural Changes in Fish Gills as Biomarker to Assess Small Stream Pollution. Tissue Cell.

[61] Yancheva V., Velcheva I., Stoyanova S., Georgieva E. (2016). Histological Biomarkers in Fish as a Tool in Ecological Risk Assessment and Monitoring Programs: A Review. Appl. Ecol. Environ. Res..

[62] Costa P.M., Diniz M.S., Caeiro S., Lobo J., Martins M., Ferreira A.M., Caetano M., Vale C., DelValls T.Á., Costa M.H. (2009). Histological Biomarkers in Liver and Gills of Juvenile *Solea senegalensis* Exposed to Contaminated Estuarine Sediments: A Weighted Indices Approach. Aquat. Toxicol..

[63] Bernet D., Schmidt H., Meier W., Burkhardt-Holm P., Wahli T. (1999). Histopathology in Fish: Proposal for a Protocol to Assess Aquatic Pollution. J. Fish Dis..

[64] Mallatt J. (1985). Fish Gill Structural Changes Induced by Toxicants and Other Irritants: A Statistical Review. Can. J. Fish. Aquat. Sci..

[65] Wegner N.C., Farrell A.P., Alderman S.L., Gillis T.E. (2024). Plasticity in Gill Morphology and Function. Encyclopedia of Fish Physiology (Second Edition).

[66] Jonz M.G. (2024). Cell Proliferation and Regeneration in the Gill. J. Comp. Physiol. B.

[67] Nilsson G.E., Dymowska A., Stecyk J.A.W. (2012). New Insights into the Plasticity of Gill Structure. Respir. Physiol. Neurobiol..

[68] Wilson J.M., Laurent P. (2002). Fish Gill Morphology: Inside Out. J. Exp. Zool..

[69] Perry S.F. (1997). The Chloride Cell: Structure and Function in the Gills of Freshwater Fishes. Annu. Rev. Physiol..

[70] Pisam M., Boeuf G., Prunet P., Rambourg A. (1990). Ultrastructural Features of Mitochondria-rich Cells in Stenohaline Freshwater and Seawater Fishes. Am. J. Anat..

[71] Pisam M., Caroff A., Rambourg A. (1987). Two Types of Chloride Cells in the Gill Epithelium of a Freshwater-adapted Euryhaline Fish: *Lebistes reticulatus*; Their Modifications during Adaptation to Saltwater. Am. J. Anat..

[72] Hwang P.-P., Lee T.-H., Lin L.-Y. (2011). Ion Regulation in Fish Gills: Recent Progress in the Cellular and Molecular Mechanisms. Am. J. Physiol.-Regul. Integr. Comp. Physiol..

[73] Dezfuli B.S., Simoni E., Giari L., Manera M. (2006). Effects of Experimental Terbuthylazine Exposure on the Cells of *Dicentrarchus labrax* (L.). Chemosphere.

[74] Fiedler S., Wünnemann H., Hofmann I., Theobalt N., Feuchtinger A., Walch A., Schwaiger J., Wanke R., Blutke A. (2020). A Practical Guide to Unbiased Quantitative Morphological Analyses of the Gills of Rainbow Trout (*Oncorhynchus mykiss*) in Ecotoxicological Studies. PLoS ONE.

[75] Uğurlu P., Satar E.İ., Çiçek T. (2019). The Histopathological, Cytopathological and Ultrastructural Effects of Carbaryl on Gills of *Oreochromis niloticus* (Linnaeus, 1758). Environ. Toxicol. Pharmacol..

[76] Loos R., Locoro G., Huber T., Wollgast J., Christoph E.H., de Jager A., Manfred Gawlik B., Hanke G., Umlauf G., Zaldívar J.M. (2008). Analysis of Perfluorooctanoate (PFOA) and Other Perfluorinated Compounds (PFCs) in the River Po Watershed in N-Italy. Chemosphere.

[77] Wei Y., Dai J., Liu M., Wang J., Xu M., Zha J., Wang Z. (2007). Estrogen-like Properties of Perfluorooctanoic Acid as Revealed by Expressing Hepatic Estrogen-Responsive Genes in Rare Minnows (*Gobiocypris rarus*). Environ. Toxicol. Chem./SETAC.

[78] Laurent P., Hoar W.S., Randall D.J. (1984). Gill Internal Morphology. Fish Physiology.

[79] Olson K.R., Alderman S.L., Gillis T.E. (2011). Branchial Anatomy. Encyclopedia of Fish Physiology (Second Edition).

[80] Monteiro S.M., Oliveira E., Fontaínhas-Fernandes A., Sousa M. (2010). Fine Structure of the Branchial Epithelium in the Teleost *Oreochromis niloticus*. J. Morphol..

[81] Kaneko T., Watanabe S., Lee K.M. (2008). Functional Morphology of Mitochondrion-Rich Cells in Euryhaline and Stenohaline Teleosts. Aqua-BioSci. Monogr. (ABSM).

[82] Pisam M. (1981). Membranous Systems in the “Chloride Cell” of Teleostean Fish Gill; Their Modifications in Response to the Salinity of the Environment. Anat. Rec..

[83] Pisam M., Rambourg A. (1991). Mitochondria-Rich Cells in the Gill Epithelium of Teleost Fishes: An Ultrastructural Approach. Int. Rev. Cytol..

[84] Komnick H., Bereiter-Hahn J., Matoltsy A.G., Richards K.S. (1986). Chloride Cells and Salt Glands. Biology of the Integument: 2 Vertebrates.

[85] Kirschner L.B. (1980). Comparison of Vertebrate Salt-Excreting Organs. Am. J. Physiol.-Regul. Integr. Comp. Physiol..

[86] Ogata T. (2000). Mammalian Tuft (Brush) Cells and Chloride Cells of Other Vertebrates Share a Similar Structure and Cytochemical Reactivities. Acta Histochem. Cytochem..

[87] Goss G., Perry S., Laurent P., Wood C.M., Shuttleworth T.J. (1995). 10 Ultrastructural and Morphometric Studies on Ion and Acid-Base Transport Processes in Freshwater Fish. Fish Physiology.

[88] Burckhardt G., Burckhardt B.C. (2011). In Vitro and in Vivo Evidence of the Importance of Organic Anion Transporters (OATs) in Drug Therapy. Drug Transporters. Handbook of Experimental Pharmacology.

[89] Hagenbuch B., Meier P.J. (2004). Organic Anion Transporting Polypeptides of the OATP/SLC21 Family: Phylogenetic Classification as OATP/SLCO Superfamily, New Nomenclature and Molecular/Functional Properties. Pflügers Arch.-Eur. J. Physiol..

[90] Gordon W.E., Espinoza J.A., Leerberg D.M., Yelon D., Hamdoun A. (2019). Xenobiotic Transporter Activity in Zebrafish Embryo Ionocytes. Aquat. Toxicol..

[91] Payan P., Girard J.P., Mayer-Gostan N., Hoar W.S., Randall D.J. (1984). 2 Branchial Ion Movements in Teleosts: The Roles of Respiratory and Chloride Cells. Fish Physiology.

[92] Dezfuli B.S., Giari L., Simoni E., Palazzi D., Manera M. (2003). Alteration of Rodlet Cells in Chub Caused by the Herbicide Stam^®^ M-4 (Propanil). J. Fish Biol..

[93] Avellán-Llaguno R.D., Liu X., Liu L., Dong S., Huang Q. (2020). Elevated Bioaccumulation of PFAAs in *Oryzias melastigma* Following the Increase of Salinity Is Associated with the up-Regulated Expression of PFAA-Binding Proteins. Sci. Total Environ..

[94] Starkov A.A., Wallace K.B. (2002). Structural Determinants of Fluorochemical-Induced Mitochondrial Dysfunction. Toxicol. Sci..

[95] Wallace K.B., Kissling G.E., Melnick R.L., Blystone C.R. (2013). Structure-Activity Relationships for Perfluoroalkane-Induced in Vitro Interference with Rat Liver Mitochondrial Respiration. Toxicol. Lett..

[96] Jung W., Park H., Lee B.-S., Chang Y.-S., Kim J.-B., Yang M.-J., Lim J., Choi H., Park E.-J. (2024). General Toxicity and Screening of Reproductive and Developmental Toxicity Following Bioaccumulation of Oral-Dosed Perfluorooctanoic Acid: Loss of the Golgi Apparatus. Food Chem. Toxicol..

[97] Sasaki K., Yoshida H. (2015). Organelle Autoregulation—Stress Responses in the ER, Golgi, Mitochondria and Lysosome. J. Biochem..

[98] Manera M., Dezfuli B.S., Castaldelli G., DePasquale J.A., Fano E.A., Martino C., Giari L. (2019). Perfluorooctanoic Acid Exposure Assessment on Common Carp Liver through Image and Ultrastructural Investigation. Int. J. Environ. Res. Public Health.

[99] Yan S., Zhang H., Wang J., Zheng F., Dai J. (2015). Perfluorooctanoic Acid Exposure Induces Endoplasmic Reticulum Stress in the Liver and Its Effects Are Ameliorated by 4-Phenylbutyrate. Free. Radic. Biol. Med..

[100] Wang Q., Chen W., Zhang B., Gao Z., Zhang Q., Deng H., Han L., Shen X.L. (2022). Perfluorooctanoic Acid Induces Hepatocellular Endoplasmic Reticulum Stress and Mitochondrial-Mediated Apoptosis in Vitro via Endoplasmic Reticulum-Mitochondria Communication. Chem.-Biol. Interact..

[101] Subash Peter M.C., Lock R.A.C., Wendelaar Bonga S.E. (2000). Evidence for an Osmoregulatory Role of Thyroid Hormones in the Freshwater Mozambique Tilapia *Oreochromis mossambicus*. Gen. Comp. Endocrinol..

[102] Peter M.C.S. (2011). The Role of Thyroid Hormones in Stress Response of Fish. Gen. Comp. Endocrinol..

[103] Yu J., Cheng W., Jia M., Chen L., Gu C., Ren H., Wu B. (2022). Toxicity of Perfluorooctanoic Acid on Zebrafish Early Embryonic Development Determined by Single-Cell RNA Sequencing. J. Hazard. Mater..

